# Reliability of the American Community Survey Estimates of Risk-Adjusted Readmission Rankings for Hospitals Before and After Peer Group Stratification

**DOI:** 10.1001/jamanetworkopen.2019.12727

**Published:** 2019-10-09

**Authors:** Nathaniel Bell, Ana Lòpez-De Fede, Bo Cai, John M. Brooks

**Affiliations:** 1 College of Nursing, University of South Carolina, Columbia; 2 Institute for Families in Society, University of South Carolina, Columbia; 3 Department of Epidemiology and Biostatistics, Arnold School of Public Health, University of South Carolina, Columbia; 4 Health Services Policy and Management, Arnold School of Public Health, University of South Carolina, Columbia

## Abstract

**Importance:**

Since the transition to the American Community Survey, data uncertainty has complicated its use for policy making and research, despite the ongoing need to identify disparities in health care outcomes. The US Centers for Medicare & Medicaid Services’ new, stratified payment adjustment method for its Hospital Readmissions Reduction Program may be able to reduce the reliance on data linkages to socioeconomic survey estimates.

**Objective:**

To determine whether there are differences in the reliability of socioeconomically risk-adjusted hospital readmission rates among hospitals that serve a disproportionate share of low-income populations after stratifying hospitals into peer group–based classification groups.

**Design, Setting, and Participants:**

This cross-sectional study uses data from the 2014 New York State Health Cost and Utilization Project State Inpatient Database for 96 278 hospital admissions for acute myocardial infarction, pneumonia, and congestive heart failure. The analysis included patients aged 18 years and older who were not transferred to another hospital, who were discharged alive, who did not leave the hospital against medical advice, and who were discharged before December 2014.

**Main Outcomes and Measures:**

The main outcomes were 30-day hospital readmissions after acute myocardial infarction, pneumonia, and congestive heart failure assessed using hierarchical logistic regression.

**Results:**

The mean (SD) age of the patients was 69.6 (16.0) years for the safety-net hospitals and 74.9 (14.7) years for the non–safety-net hospitals; 9382 (48.8%) and 7003 (48.5%) patients, respectively, were female. For safety net designations, 20% (3 of 15) of all evaluations concealed and distorted differences in risk, with factors such as poverty failing to identify similar risk of acute myocardial infarction readmission until unreliable estimates were excluded from the analysis (OR, 1.23 [95% CI, 1.00-1.52], *P* = .02; vs OR, 1.17 [95% CI, 0.94-1.46], *P* = .15). By comparison, 2 of the 60 models (3%) for the peer group–based classification altered the association between socioeconomic status and readmission risk, concealing similarities in congestive heart failure readmission when adjusted using high school completion rates (OR, 1.27 [95% CI 1.02-1.58], *P* = .04; vs OR, 1.23 [95% CI, 0.98-1.53], *P* = .06) and distorting similarities in pneumonia readmissions when accounting for the proportion of lone-parent families (OR, 1.27 [95% CI, 0.98-1.66], *P* = .07; vs OR, 1.35 [95% CI, 1.02-1.80], *P* = .04) between the lowest and highest socioeconomic status hospitals in quartile 1.

**Conclusions and Relevance:**

There was greater precision in socioeconomic adjusted readmission estimates when hospitals were stratified into the new payment adjustment criteria compared with safety net designations. A contributing factor for improved reliability of American Community Survey estimates under the new payment criteria was the merging of patients from low-income neighborhoods with greater homogeneity in survey estimates into groupings similar to those for higher-income patients, whose neighborhoods often exhibit greater estimate variability. Additional efforts are needed to explore the effect of measurement error on American Community Survey–adjusted readmissions using the new peer group–based classification methods.

## Key Points

**Question:**

Is the quality of American Community Survey estimates associated with changes in hospital readmission rankings after socioeconomic risk adjustment?

**Findings:**

In this cross-sectional study of 96 278 hospital admissions for acute myocardial infarction, pneumonia, and congestive heart failure included in the 2014 New York State Health Cost and Utilization Project, compared with previous stratification by safety-net hospital designation, the new peer group–based stratification system was associated with improved reliability of American Community Survey socioeconomic status estimates.

**Meaning:**

Poor reliability in the American Community Survey can conceal and distort readmission rates for hospitals, and the use of peer group–based performance measurement is associated with a reduction, but not elimination, of the impact of measurement error on risk-adjusted rates.

## Teaser

This cross-sectional study uses data from the New York State Health Cost and Utilization Project State Inpatient Database to examine whether the reliability of American Community Survey socioeconomic status estimates improves with the use of new peer group–based strata compared with previous stratification by safety-net hospital designation.

## Introduction

Adhering to the Centers for Medicare & Medicaid Services (CMS) Hospital Readmissions Reduction Program (HRRP) performance and accountability metrics presents a profound and ongoing challenge for many safety-net hospitals (SNHs).^1,2,3^ Because these hospitals care for a disproportionate number of socially and economically vulnerable patients, their readmission rates are often higher than those of non-SNHs, driven by challenges brought on by poverty, low health literacy, poor housing conditions, and a lack of social support and access to care.^4,5,6,7,8^ These conditions often coincide with the primary reasons most patients cite as contributing to relapse and readmission.^9^ In particular, research^10^ indicates that differences in hospital readmission rates between SNHs and non-SNHs often become negligible after accounting for patient socioeconomic position, or socioeconomic status (SES), thus emphasizing the substantive association between the socioeconomic case mix of a hospital’s patient population and interpretations of its quality and performance.

In an attempt to address this concern, CMS recently (fiscal year 2019) introduced a new peer group–based payment adjustment method into the HRRP to account for differences in readmission risk attributed to differences in patient socioeconomic case mix.^11^ Because hospitals lack individual-level data on patient socioeconomic case mix, CMS chose to classify institutions according to their proportion of fee-for-service Medicare and Medicare Advanced hospitalizations, for which the patient is eligible for both Medicare and Medicaid reimbursement. Initial evaluations of the new payment model suggest that a peer group–based method measured at the hospital level offers some reprieve to SNHs but does not eliminate the cost imbalances associated with the disproportionate burden these facilitates experience because they serve low-income populations.^12^

To our knowledge, there has been no evaluation of CMS’s new payment model structure relative to other measures of SES. Given the ever-changing insurance situation in the United States, insurance categories may not consistently be associated with income and other socioeconomic measures. However, because hospitals typically do not collect patient-level socioeconomic data, any measure of SES beyond insurance status must be derived by geocoding hospitalization records to the US Census. This approach is problematic because it attempts to infer specific meaning from individual events that are drawn from aggregate data. Because of this reliance, a second and potentially more profound challenge has now emerged. At issue is that the recent transition from the long-form decennial US Census to the American Community Survey (ACS) fundamentally changed the quality of demographic, socioeconomic, and housing data collected from the population. For example, some studies^13^ have found the margins of error in ACS poverty estimates to be so large that a neighborhood can switch from the least to the most deprived SES quartile. Accordingly, in this study, we tested whether proxy measures of patient SES were significantly associated with readmission risk before and after the switch to the peer group–based stratification model. Our primary focus was whether the reliability of the ACS estimates was associated with instances where patients’ SES explained the differences in readmission risk.

## Methods

### Data Source

This cross-sectional study was conducted using the 2014 New York State Health Cost and Utilization Project State Inpatient Database (SID) discharge records and corresponding SES estimates from the 2014 ACS 5-year zip code–tabulated area data cycles that matched the patient’s place of residence (not the hospital’s location). In 2014, New York was 1 of 13 states that submitted data to the SID that also contained identifiers that can be used to identify 30-day hospital readmissions.^14^ Not all states that contribute to the SID readmission file submit geographic identifiers.

This study was reviewed by the University of South Carolina institutional review board and was deemed to have met the not human research criteria set forth by the Common Rule (45CFR46). This study follows the Strengthening the Reporting of Observational Studies in Epidemiology (STROBE) reporting guideline.

### Study Population and Outcomes

We evaluated readmissions for acute myocardial infarction (AMI), pneumonia, and congestive heart failure (CHF) because these were the 3 medical conditions linked to HRRP payments during this year. Each patient panel was defined using the *International Classification of Diseases*, *Ninth Revision*, *Clinical Modification* codes using the same codes provided in the CMS readmission reports.^15,16,17^ All evaluations were based on the index hospitalization, and only the first readmission was counted.

We used Agency for Healthcare Research and Quality criteria^18^ to define SNHs as the top quartile of hospitals having the highest number of inpatient stays among Medicaid or uninsured recipients. Safety-net hospitals were defined using the entire patient sample in the SID. The CMS’s new group-based criteria stratify hospitals into quintiles on the basis of the proportion of dually eligible Medicare and full-benefit Medicaid status. To replicate this method, we extrapolated the expected payment status from primary and secondary payer fields in the SID. The SID payer fields do not differentiate between fee-for-service or managed care patients. We grouped hospitals by dual Medicare and Medicaid enrollment into quartiles to draw comparisons with established SNH classifications. Similar to the current and previous payment methods, we limited our analysis to hospitals with 25 or more eligible discharges for each condition.

All models were adjusted for patient age, sex, and comorbid health status, as well as 5 SES measures: no high school completion, median household income, female lone-parent families, area poverty rates (all persons), and the area unemployment rate. Each measure was selected for analysis because of theoretical and empirical evidence linking it with increased hospital readmissions. All measures were constructed using zip codes because this is the finest spatial resolution available in the Health Cost and Utilization Project SID for data linkage. Although zip codes can be less homogeneous than US Census Tract and Block groups, they typically produce similar, although attenuated, associations with health disparities.^19^ Patient health status was measured using the Elixhauser software distributed by the Agency for Healthcare Research and Quality,^20^ because of its frequent use in hospital risk adjustment analyses owing to a lack of outpatient claims data.^10,21^

### Socioeconomic Status Estimation

We expressed the relative uncertainty for each ACS estimate by following US Census Bureau guidelines for calculating its coefficient of variation (CV) and margin of error.^22^ The margin of error for unemployment status was calculated using guidelines for derived proportions. The measure of educational attainment and female lone-parent families required multiple numerators or denominators to build the proportions. For these, the numerators and denominators were aggregated within the equation for calculating the margin of error for the derived estimate. Median household income and poverty status did not require further calculation because these estimates are published as percentages or monetary values. We used an adjustment factor by multiplying the margin of error for each estimate or its numerator or denominator by a factor equal to 1.960 or 1.64 to derive the SE for each estimate with 95% confidence.

The National Research Council^23^ defines a reasonable standard of precision for each ACS estimate as a CV score less than 12%. Categorical rankings of CV precision are also defined as high reliability when CV scores are less than 12%, moderate reliability for CV scores between 12% and 40%, and low or unreliable when CV scores exceed 40%.^24^ The process required for evaluating the precision of the ACS estimates is described in greater detail by the US Census Bureau.^25^ We used US Census Bureau thresholds for defining estimates as either reliable (CV ≤40%) or unreliable (CV >40%). The CV scores are often used as litmus test for the ACS because they allow uncertainty to be expressed in relative terms (eg, a CV score of 50% for a median household income of $50 000 would be ± $25 000 in the estimate). We excluded homeless populations from all analyses because they lack a geographic identifier that can link them to US Census data, the lack of a geographic identifier makes it infeasible to test estimate reliability, and the definition of homelessness can vary substantially across health care systems. More than 96% of patients defined as homeless in the New York SID were treated for AMI, CHF, or pneumonia by SNHs.

### Statistical Analysis

The primary objective of our analysis was to assess whether ACS reliability was associated with the global interpretation of SES-adjusted 30-day readmission risk when institutions were defined as SNHs compared with the new CMS peer group–based method. For our first test, we used a hierarchical logistical regression model to estimate the independent association between risk-adjusted 30-day readmission rates and hospital grouping designation after adjusting for patient age, sex, and health status using the Elixhauser algorithm. We then extended this model to include adjustment for SES regardless of the estimate’s precision. In our last test, we limited the analysis to adjustment using only reliable estimates. Using an approach similar to the method used by CMS for its readmission rankings, we estimated readmission risk using hierarchical logistic regression specifying hospitals as a random effect to account for clustering.

Social, economic, and demographic factors are perceived to be associated with readmission risk if differences between facilities become negligible after their inclusion in the adjustment model.^26,27^ We inferred that measurement reliability had an impact on this association if the removal of unreliable estimates altered the observed effect. We used adjusted odds ratios (ORs) to assess whether measurement error was associated with the likelihood that a patient would be readmitted to an SNH, compared with patients in non-SNHs. For the SNH analysis, non-SNH hospitals (lowest quartile) were the reference category. For the peer-group analysis, the highest-SES hospitals in each stratum were the reference group. The analysis included patients aged 18 years and older who were not transferred to another hospital, who were discharged alive, who did not leave the hospital against medical advice, and who were discharged before December 2014. All models were run using the GLIMMIX procedure in SAS statistical software version 9.4 for Windows (SAS Institute) using maximum likelihood estimation based on Laplace approximation. Two-sided *P* < .05 was considered significant.

## Results

As shown in the Figure, approximately 40% of ACS zip code estimates for high school completion, female lone-parent families, poverty, and unemployment for the state of New York are considered to be unreliable according to their CV scores. Median household income was the most reliable measure in all comparisons. The Figure also illustrates that when SES scores were stratified by hospital grouping designation, SNHs served patients who had more reliable SES data. For all measures, no more than 1% of the SES estimates for SNH patients had levels of error that were deemed to be unreliable. By comparison, approximately 11% of estimates assigned to non-SNHs could be considered too imprecise to use. These trends remained when assessed across specific AMI, pneumonia, and CHF panels (data not shown). In contrast, the Figure also shows there was little discernable difference in the proportion of unreliable data assigned to each patient when hospitals were stratified using CMS’s new peer group–based criteria. By comparison, only 11 of the 207 institutions defined as SNHs were similarly grouped into CMS’s lowest-SES peer group (21.2%), whereas 41.5% of SNH hospitals were designated into the highest SES peer group (χ^2^ = 20.78; *P* = .01). Given the differences in estimate reliability across hospital classification groups, we examined SES-adjusted readmission risk before and after accounting for the precision of each measure.

**Figure.**
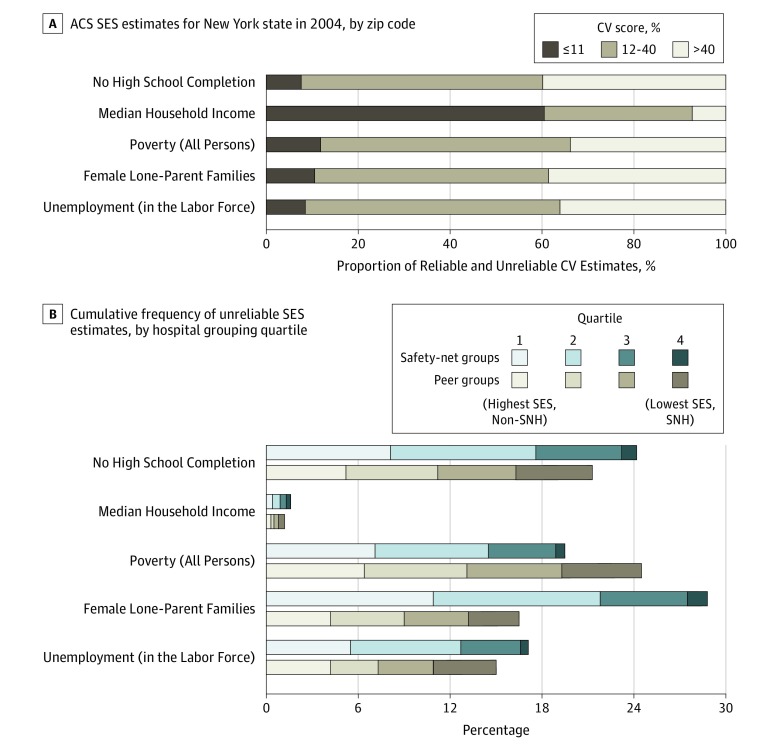
Reliability of American Community Survey (ACS) Socioeconomic Status (SES) Estimates and Cumulative Frequency of Unreliable Estimates A, Proportions of reliable and unreliable SES estimates owing to sampling errors in the ACS SES estimates released for New York State for the 2014 five-year data cycle are shown by zip code. Reliability was measured using margins of errors and coefficient of variation (CV) with 95% confidence limits, with CV scores of up to 11% and 12% to 40% considered reliable, and scores greater than 40% considered unreliable. B, The cumulative distribution of unreliable SES estimates (CV score >40%) across different hospital grouping quartiles is also shown. SNH indicates safety-net hospital.

Table 1 shows sample sizes and patient clinical and demographic characteristics within each hospital grouping. The mean (SD) age of the patients was 69.6 (16.0) years for the SNHs and 74.9 (14.7) years for the non-SNHs; 9382 (48.8%) and 7003 (48.5%) patients, respectively, were female. The mean (SD) age within the lowest SES peer group was 72.0 (15.2) years, compared with 70.8 (15.6) years within the highest SES group; 8931 (50.0%) and 9055 (49.4%) of patients in each group, respectively, were female. Readmission rates for AMI were 18.3% for SNHs vs 17.0% for non-SNHs, rates for pneumonia were 16.5% for SNHs vs 17.1% for non-SNHs, and rates for CHF were 27.4% for SNHs vs 23.9% for non-SNHs. Baseline estimates and event counts within each strata and the association of adjustment for patient age, sex, and comorbid conditions with readmission rates before SES adjustment are shown in Table 2. For example, for all SNHs (those stratified into quartile 4), odds of readmissions were statistically significantly higher for AMI (OR, 1.33; 95% CI, 1.08-1.64; *P* = .01) and CHF (OR, 1.17; 95% CI, 1.04-1.33; *P* = .01) compared with non-SNHs, but not for pneumonia readmissions (OR, 1.09; 95% CI, 0.93-1.28; *P* = .31). When stratified using the group-based criteria, hospitals classified into the lowest SES category in quartile 4 (ie, lowest SES group) and hospitals classified into the lowest SES category within quartile 1 (ie, highest SES group) generated statistically significant differences in readmission risk before SES adjustment. For both quartile 4 and quartile 1, the lowest SES hospitals had significantly higher odds of CHF readmission risk, ranging from an OR of 1.29 (95% CI, 1.04-1.61; *P* = .02) for quartile 4 to an OR of 1.46 (95% CI, 1.12-1.92; *P* = .01) for hospitals in quartile 1.

**Table 1.** Patient Characteristics by Hospital Designation Group, 2014 New York Health Cost and Utilization Project State Inpatient Database

Characteristic	SNH^a^	Non-SNH^a^	Stratification Based on Hospital Proportion of Dually Enrolled Medicare and Medicaid Populations^b^			
			Quartile 4, Lowest SES	Quartile 3	Quartile 2	Quartile 1, Highest SES
Patients, No.	13 282	80 996	17 869	32 144	25 931	18 334
Age, mean (SD), y	69.6 (16.0)	74.9 (14.7)	72.0 (15.2)	71.2 (15.6)	72.1 (15.4)	70.8 (15.6)
Female, %	48.8^c^	48.5^c^	50.0	49.0	45.9	49.4
Race/ethnicity, %						
White	26.6	79.1	60.9	56.4	69.5	67.2
Black	32.0	7.1	19.8	17.2	12.6	15.9
Hispanic	21.8	3.2	8.1	11.9	5.9	11.0
Other	19.6	10.5	11.2	14.5	12.0	5.9
Insurance type, %						
Private	11.3	17.2	13.0	14.1	19.2	14.3
Medicare	61.5	75.0	70.1	69.3	68.7	68.7
Medicaid	22.5	4.8	14.2	14.4	8.1	12.3
Self-pay	3.7	1.0	1.6	1.4	2.2	2.5
Other	1.0	2.0	1.1	0.7	1.8	2.3
Urgency, %						
Emergency	95.7	88.3	88.8	86.9	88.6	88.6
Urgent	2.3	8.1	8.2	10.0	6.9	7.2
Elective	2.0	3.6	2.9	3.1	4.4	4.2
Readmission rate by medical condition, %						
Acute myocardial infarction	18.3	17.0	17.4^c^	17.3^c^	16.1^c^	17.0^c^
Pneumonia	16.5	17.1	18.2^c^	17.8^c^	17.3^c^	18.0^c^
Congestive heart failure	27.4	23.9	26.8	25.4	24.9	25.3
Agency for Healthcare Research and Quality comorbidity measures, %						
Congestive heart failure	55.3	53.9	50.6	53.7	53.8	53.7
Valvular disease	14.3	26.4	15.8	19.7	23.8	19.5
Pulmonary circulation disease	11.7	16.0	10.5	13.5	15.7	12.6
Peripheral vascular disease	7.6	11.2	9.3	9.0	10.4	10.1
Paralysis	3.1	2.5	3.1	2.7	2.0	2.4
Other neurological disorders	7.9	8.8	10.3	8.1	7.9	8.8
Chronic pulmonary disease	31.7	36.3	38.9	34.6	33.0	35.9
Diabetes without chronic complications	35.2	28.7	33.4	32.9	30.1	30.9
Diabetes with chronic complications	9.3	7.5	7.6	8.2	6.8	9.3
Hypothyroidism	11.1	18.6	15.3	14.8	16.4	16.4
Renal failure	31.5	29.2	30.0	30.9	29.2	33.0
Liver disease	4.1	2.4	2.7	2.9	2.7	3.1
Peptic ulcer disease with bleeding	0.1	0.0	0.1^c^	0.1^c^	0.0^c^	0.1^c^
AIDS	0.0	0.0	0.0	0.0	0.0	0.0
Lymphoma	1.2	1.9	12.4	1.4	1.8	1.4
Metastatic cancer	1.7	3.5	1.8	2.1	2.6	1.9
Solid tumor without metastasis	2.7	2.9	2.6	2.7	2.4	2.9
Rheumatoid arthritis or collagen vascular disease	2.2	3.5	2.7	3.2	3.5	3.4
Coagulopathy	4.9	6.0	4.6	5.0	6.0	5.9
Obesity	15.4	12.8	15.3	15.3	13.3	17.4
Weight loss	3.8	3.4	4.8	3.9	4.1	3.7
Fluid and electrolyte disorders	27.3	32.8	31.6	29.4	30.6	31.5
Chronic blood loss anemia	0.5	0.7	0.7	0.7	0.5	0.8
Deficiency anemias	24.5	24.1	25.0	24.5	22.5	26.6
Alcohol abuse	4.8	2.0	3.3	3.0	2.6	3.8
Drug abuse	5.8	1.1	2.5	3.1	2.2	3.5
Psychoses	4.0	2.6	4.2	3.5	3.0	3.8
Depression	7.7	12.3	10.9	10.2	11.6	12.5
Hypertension	76.8	72.3	75.0^c^	74.6^c^	74.6^c^	75.2^c^
Proportion of patients in the most deprived quartile, %						
No high school completion	81.9	23.1	52.2	43.5	30.1	41.5
Median household income	56.5	14.1	41.7	30.8	18.5	40.5
Poverty (all persons)	71.0	17.7	50.3	43.3	24.6	43.3
Female lone-parent families	62.9	18.0	42.5	40.0	24.2	47.4
Unemployment (in the labor force)	56.4	15.9	32.7	31.4	20.5	33.4
Estimates with coefficient of variation values >40%, %						
No high school completion	1.0	7.8	6.5	5.6	7.2	6.3
Median household income	0.0	0.4	0.5	0.3	0.3	0.4
Poverty (all persons)	0.6	6.9	4.0	4.7	6.1	5.1
Female lone-parent families	1.3	10.4	6.6	7.0	7.7	8.5
Unemployment (in the labor force)	0.5	5.3	5.1	4.4	4.2	5.8

Abbreviations: SES, socioeconomic status; SNH, safety-net hospital.

^a^ An SNH is defined as having 38.7% to 96.7% of patients enrolled in Medicaid, and a non-SNH is defined as having 2.0% to 38.6% of patients enrolled in Medicaid.

^b^ The proportions of patients dually enrolled in Medicare and Medicaid are 0.0% to 3.9% for quartile 1 hospitals, 7.3% to 5.8% for quartile 2 hospitals, 7.4% to 10.8% for quartile 3 hospitals, and 11.0% to 16.6% for quartile 4 hospitals.

^c^ Statistically significant (*P* < .05).

**Table 2.** Association of Hospital Grouping Classification of Economically Disadvantaged Patients With 30-Day Hospital Readmissions, 2014 New York Health Cost and Utilization Project State Inpatient Database

Condition and Characteristics	SNH^a^		Stratification Based on Hospital Proportion of Dually Enrolled Medicare and Medicaid Populations^b^							
			Quartile 4, Lowest SES		Quartile 3		Quartile 2		Quartile 1, Highest SES	
	OR (95% CI)	*P* Value	OR (95% CI)	*P* Value	OR (95% CI)	*P* Value	OR (95% CI)	*P* Value	OR (95% CI)	*P* Value
Acute myocardial infarction										
Quartile 1, highest SES	1 [Reference]		1 [Reference]		1 [Reference]		1 [Reference]		1 [Reference]	
Quartile 2	1.07 (0.89-1.29)	.48	1.52 (1.06-1.71)	.03	0.97 (0.80-1.18)	.72	0.93 (0.68-1.29)	.67	1.11 (0.67-1.84)	.69
Quartile 3	1.20 (0.99-1.46)	.06	1.25 (0.92-1.71)	.14	1.20 (0.93-1.55)	.16	1.04 (0.76-1.40)	.82	1.02 (0.50-2.06)	.97
Quartile 4, lowest SES	1.33 (1.08-1.64)	.01	1.34 (0.90-1.98)	.14	1.05 (0.85-1.29)	.64	0.91 (0.65-1.28)	.59	1.52 (0.89-2.59)	.12
Hospitals, No.	150		29		37		47		37	
Nonreadmissions, No.	18 212		2541		5607		6183		2881	
Readmissions, No.	3701		727		1077		1290		607	
Pneumonia										
Quartile 1, highest SES	1 [Reference]		1 [Reference]		1 [Reference]		1 [Reference]		1 [Reference]	
Quartile 2	1.11 (0.96-1.27)	.16	1.02 (0.75-1.38)	.90	0.89 (0.66-1.20)	.44	0.87 (0.69-1.09)	.22	0.98 (0.73-1.33)	.92
Quartile 3	1.17 (1.01-1.34)	.04	1.22 (0.95-1.58)	.12	0.84 (0.61-1.16)	.28	1.01 (0.80-1.27)	.93	1.09 (0.79-1.49)	.59
Quartile 4, lowest SES	1.09 (0.93-1.28)	.31	1.26 (0.96-1.65)	.10	1.12 (0.83-1.50)	.45	0.90 (0.70-1.15)	.38	1.06 (0.79-1.42)	.71
Hospitals, No.	180		38		42		51		49	
Nonreadmissions, No.	24 759		4841		6497		8225		5196	
Readmissions, No.	5356		1059		1362		1779		1156	
Congestive heart failure										
Quartile 1, highest SES	1 [Reference]		1 [Reference]		1 [Reference]		1 [Reference]		1 [Reference]	
Quartile 2	1.02 (0.91-1.14)	.74	1.34 (1.07-1.69)	.01	1.13 (0.96-1.34)	.14	0.89 (0.74-1.07)	.20	1.07 (0.82-1.40)	.62
Quartile 3	1.06 (0.94-1.20)	.33	1.23 (1.00-1.52)	.05	1.16 (0.97-1.38)	.10	0.99 (0.82-1.18)	.88	1.49 (1.11-2.00)	.01
Quartile 4, lowest SES	1.17 (1.04-1.33)	.01	1.29 (1.04-1.61)	.02	1.10 (0.93-1.31)	.26	0.99 (0.82-1.19)	.89	1.46 (1.12-1.92)	.01
Hospitals, No.	178		37		43		51		47	
Nonreadmissions, No.	31 471		6104		8552		10 937		5878	
Readmissions, No.	10 779		2062		2836		3730		2151	

Abbreviations: OR, odds ratio; SES, socioeconomic status; SNH, safety-net hospital.

^a^ An SNH is defined as having 38.7% to 96.7% of patients enrolled in Medicaid.

^b^ The proportions of patients dually enrolled in Medicare and Medicaid are 0.0% to 3.9% for quartile 1 hospitals, 7.3% to 5.8% for quartile 2 hospitals, 7.4% to 10.8% for quartile 3 hospitals, and 11.0% to 16.6% for quartile 4 hospitals.

Without accounting for SES, the new classification groups generated fewer numbers of penalties compared with SNH designation, ranging from 1.0% (1 penalty) based on peer group–based rankings compared with 2.7% (4 penalties) among SNHs for AMI readmissions, from 3.3% (6 penalties) to 8.9% (16 penalties) for pneumonia readmissions, and from 2.2% (4 penalties) to 6.2% (11 penalties) for CHF readmissions, respectively. In total, we identified 2 SNH hospitals that would have potentially been penalized under the HRRP’s old ranking criteria, whereas 1 institution in peer group quartile 4 would have been similarly eligible. Overall, 12 unique hospitals designated under the old penalty model were potentially eligible for penalty compared with 3 institutions under the new payment model.

Table 3 shows that after accounting for the quality of area poverty rates, differences in AMI readmissions could have been 6% lower among SNHs compared with non-SNHs, a negative association, or up to 46% higher, a positive association (OR, 1.23 [95% CI, 1.00-1.52], *P* = .02 vs OR, 1.17 [95% CI, 0.94-1.46], *P* = .15). Without the exclusion of unreliable poverty estimates, odds of AMI readmissions among SNHs would traditionally have been interpreted as 3% to 59% higher despite accounting area poverty rates (OR, 1.28; 95% CI, 1.03-1.59; *P* = .03). The ORs of readmission for AMI were 1.19 (95% CI, 0.95-1.48; *P* = .12) after adjustment for high school completion rate, 1.26 (95% CI, 1.02-1.56; *P* = .03) for median household income, 1.14 (95% CI, 0.92-1.41; *P* = .24) for female lone-parent families, and 1.26 (95% CI, 1.02-1.57; *P* = .04) for area unemployment rates. Similar differences in association between SES and readmission risk were observed for CHF readmissions when adjusted using area income rates. In this instance, differences in risk could have been 3% lower in SNHs or up to 29% higher after accounting for SES (OR, 1.13; 95% CI, 1.00-1.29; *P* = .06). Before excluding unreliable estimates, CHF readmission rates would have been interpreted as less than or equal to 29% higher even after accounting for median income (OR, 1.14; 95% CI, 1.00-1.29; *P* = .05). In other comparisons for CHF, data errors in high school completion rates exaggerated the equalizing effect of SES adjustment on differences in readmissions (OR, 1.15; 95% CI, 1.02-1.31; *P* = .03), whereas neither area unemployment rates (OR, 1.15; 95% CI, 1.02-1.31; *P* = .03) nor rates of female lone-parent families (OR, 1.13; 95% CI, 0 .99-1.28; *P* = .06) removed overall differences in 30-day readmission rates for SNHs. For pneumonia readmissions, ORs were 1.08 (95% CI, 0.91-1.28; *P* = .36) after adjustment for high school completion rates, 1.06 (95% CI, 0.91-1.25; *P* = .45) after adjustment for median household income, 1.07 (95% CI, 0.91-1.26; *P* = .42) after adjustment for poverty, 1.07 (95% CI, 0.90-1.26; *P* = .45) after adjustment for female lone-parent families, and 1.09 (95% CI, 0.92-1.29; *P* = .32) after adjustment for area unemployment rates. In total, 3 of the 15 SES models based on SNH designation (20%) were susceptible to error due to the inclusion of ACS SES estimates.

**Table 3.** Association of American Community Survey Estimate Reliability With Safety-Net Hospital Readmission Risk

Variable	All SES Estimates		Excluding CV >40%	
	OR (95% CI)	*P* Value	OR (95% CI)	*P* Value
Acute myocardial infarction readmissions				
No high school completion	1.27 (1.03-1.57)	.10	1.19 (0.95-1.48)	.12
Median household income	1.28 (1.03-1.59)	.03	1.26 (1.02-1.56)	.03
Poverty (all persons)	1.23 (1.00-1.52)	.02	1.17 (0.94-1.46)	.15
Female lone-parent families	1.28 (1.04-1.58)	.05	1.14 (0.92-1.41)	.24
Unemployment (in the labor force)	1.08 (0.91-1.26)	.02	1.26 (1.02-1.57)	.04
Pneumonia readmissions				
No high school completion	1.06 (0.91-1.25)	.38	1.08 (0.91-1.28)	.36
Median household income	1.05 (0.90-1.24)	.45	1.06 (0.91-1.25)	.45
Poverty (all persons)	1.06 (0.90-1.24)	.54	1.07 (0.91-1.26)	.42
Female lone-parent families	1.09 (0.92-1.27)	.50	1.07 (0.90-1.26)	.45
Unemployment (in the labor force)	1.13 (0.99-1.28)	.32	1.09 (0.92-1.29)	.32
Congestive heart failure readmissions				
No high school completion	1.13 (0.99-1.28)	.06	1.15 (1.02-1.31)	.03
Median household income	1.14 (1.00-1.29)	.05	1.13 (1.00-1.29)	.06
Poverty (all persons)	1.13 (0.99-1.28)	.06	1.13 (0.99-1.29)	.06
Female lone-parent families	1.13 (1.00-1.29)	.05	1.13 (0.99-1.28)	.06
Unemployment (in the labor force)	1.15 (1.01-1.30)	.03	1.15 (1.02-1.31)	.03

Abbreviations: CV, coefficient of variation; OR, odds ratio; SES, socioeconomic status.

Table 4 extends the results shown for SNHs to the recently modified hospital peer group–based grouping criteria. The results shown in Table 4 contrast hospitals grouped into the lowest-SES quartile with those in the highest-SES quartile across all strata. Across the 60 comparisons, there were 2 instances (3%) where the exclusion of unreliable SES estimates altered the association between SES and readmission risk, specifically readmission rates for pneumonia in quartile 1 for female lone-parent families (OR, 1.27 [95% CI, 0.98-1.66], *P* = .07; vs 1.35 [95% CI, 1.02-1.80], *P* = .04) and for readmission rates for CHF in quartile 1 for no high school completion (OR, 1.27 [95% CI, 1.02-1.58], *P* = .04; vs OR, 1.23 [95% CI, 0.98-1.53], *P* = .06). With the exception of adjustment for area poverty and high school completion rates, SES did not alter differences in readmission risk between hospitals classified into the lowest SES quartile compared with the reference group for any medical condition other than CHF. Differences in CHF readmissions among hospitals classified into the lowest SES quartile were not explained by high school completion rates (OR, 1.37; 95% CI, 1.04-1.80; *P* = .03), median household income (OR, 1.47; 95% CI, 1.11-1.95; *P* = .01), poverty (OR, 1.40; 95% CI, 1.05-1.85; *P* = .02), lone-parent families (OR, 1.35; 95% CI, 1.05-1.74; *P* = .02), or area unemployment rates (OR, 1.39; 95% CI, 1.06-1.82; *P* = .01). After removing unreliable estimates, adjustment for area poverty rates removed differences in readmission risk within hospitals classified into the lowest SES group within quartile 1 compared with the unadjusted model (OR, 1.24 [95% CI, 0.99-1.54], *P* = .06 vs 1.29 [95% CI, 1.04-1.61], *P* = .02). Similarly, Table 4 also shows that after accounting for the quality of area high school completion rates, differences in CHF readmission rates could have been 2% lower among hospitals in the lowest SES classification within quartile 1, a small negative association, whereas without excluding unreliable estimates, readmission risk would have found a small positive association. In all comparisons, the total number of patients excluded from analysis after eliminating unreliable US Census estimates was less than 5%.

**Table 4.** Association of American Community Survey Estimate Reliability With Peer Group–Based Stratified Readmission Risk

Variable	Quartile 1, Highest SES				Quartile 2				Quartile 3				Quartile 4, Lowest SES			
	All SES Estimates		Excluding CV >40%		All SES Estimates		Excluding CV >40%		All SES Estimates		Excluding CV >40%		All SES Estimates		Excluding CV >40%	
	OR (95% CI)	*P* Value	OR (95% CI)	*P* Value	OR (95% CI)	*P* Value	OR (95% CI)	*P* Value	OR (95% CI)	*P* Value	OR (95% CI)	*P* Value	OR (95% CI)	*P* Value	OR (95% CI)	*P* Value
Acute myocardial infarction readmissions																
No high school completion	1.36 (0.94-1.96)	.10	1.41 (0.98-2.05)	.07	1.05 (0.85-1.28)	.66	1.05 (0.85-1.30)	.64	0.88 (0.64-1.21)	.42	0.85 (0.62-1.16)	.31	1.46 (0.85-2.51)	.16	1.48 (0.85-2.60)	.16
Median household income	1.36 (0.92-1.97)	.12	1.35 (0.92-1.97)	.12	1.02 (0.81-1.28)	.87	1.03 (0.82-1.28)	.82	0.92 (0.65-1.30)	.62	0.92 (0.65-1.29)	.61	1.49 (0.86-2.58)	.15	1.47 (0.85-2.55)	.16
Poverty (all persons)	1.34 (0.92-1.95)	.12	1.35 (0.91-2.01)	.13	1.05 (0.85-1.30)	.66	1.00 (0.80-1.25)	.99	0.91 (0.65-1.28)	.58	0.89 (0.64-1.24)	.47	1.53 (0.89-2.63)	.12	1.51 (0.84-2.71)	.16
Female lone-parent families	1.37 (0.98-1.93)	.07	1.39 (0.95-2.03)	.09	1.04 (0.81-1.31)	.77	1.03 (0.82-1.28)	.82	0.91 (0.66-1.26)	.56	0.90 (0.65-1.25)	.52	1.50 (0.88-2.56)	.13	1.53 (0.85-2.76)	.15
Unemployment (in the labor force)	1.32 (0.90-1.95)	.15	1.40 (0.95-2.05)	.09	1.04 (0.85-1.28)	.68	1.00 (0.83-1.22)	.97	0.91 (0.66-1.26)	.57	0.90 (0.66-1.24)	.52	1.43 (0.85-2.41)	.17	1.30 (0.73-2.30)	.36
Pneumonia readmissions																
No high school completion	1.28 (0.97-1.69)	.08	1.29 (0.98-1.7)	.06	1.12 (0.83-1.49)	.45	1.12 (0.83-1.51)	.44	0.91 (0.70-1.17)	.43	0.92 (0.71-1.20)	.53	1.07 (0.79-1.44)	.65	1.13 (0.81-1.57)	.47
Median household income	1.28 (0.98-1.68)	.07	1.30 (0.99-1.71)	.06	1.11 (0.83-1.49)	.46	1.12 (0.84-1.50)	.44	0.91 (0.71-1.16)	.45	0.91 (0.71-1.16)	.45	1.06 (0.78-1.43)	.71	1.07 (0.79-1.45)	.66
Poverty (all persons)	1.24 (0.95-1.62)	.11	1.29 (0.99-1.68)	.06	1.10 (0.82-1.48)	.51	1.04 (0.77-1.41)	.80	0.90 (0.70-1.15)	.39	0.93 (0.72-1.19)	.55	1.05 (0.78-1.41)	.76	1.08 (0.78-1.49)	.64
Female lone-parent families	1.27 (0.98-1.66)	.07	1.35 (1.02-1.80)	.04	1.10 (0.82-1.47)	.53	1.07 (0.80-1.43)	.66	0.91 (0.71-1.17)	.46	0.92 (0.72-1.18)	.51	1.05 (0.78-1.41)	.74	1.02 (0.74-1.39)	.92
Unemployment (in the labor force)	1.27 (0.97-1.67)	.10	1.32 (0.99-1.78)	.06	1.11 (0.83-1.49)	.47	1.07 (0.79-1.45)	.67	0.92 (0.72-1.17)	.50	0.91 (0.73-1.15)	.44	1.06 (0.79-1.43)	.69	1.08 (0.78-1.49)	.65
Congestive heart failure readmissions																
No high school completion	1.27 (1.02-1.58)	.04	1.23 (0.98-1.53)	.06	1.09 (0.92-1.29)	.34	1.14 (0.96-1.36)	.14	0.93 (0.79-1.11)	.42	0.96 (0.81-1.13)	.56	1.41 (1.07-1.85)	.02	1.37 (1.04-1.80)	.03
Median household income	1.28 (1.03-1.60)	.03	1.28 (1.02-1.59)	.03	1.07 (0.90-1.28)	.42	1.17 (0.90-1.28)	.45	0.96 (0.79-1.17)	.67	0.96 (0.80-1.16)	.66	1.47 (1.11-1.95)	.01	1.47 (1.11-1.95)	.01
Poverty (all persons)	1.24 (0.99-1.54)	.06	1.25 (0.99-1.57)	.06	1.07 (0.90-1.28)	.42	1.08 (0.91-1.30)	.36	0.95 (0.79-1.14)	.58	0.96 (0.80-1.17)	.67	1.43 (1.08-1.89)	.01	1.40 (1.05-1.85)	.02
Female lone-parent families	1.29 (1.03-1.62)	.03	1.32 (1.04-1.66)	.02	1.09 (0.92-1.30)	.32	1.12 (0.94-1.34)	.19	0.98 (0.82-1.17)	.79	0.98 (0.83-1.16)	.86	1.45 (1.11-1.88)	.01	1.35 (1.05-1.74)	.02
Unemployment (in the labor force)	1.27 (1.02-1.57)	.03	1.32 (1.05-1.66)	.02	1.09 (0.92-1.29)	.33	1.10 (0.93-1.31)	.25	0.97 (0.81-1.16)	.74	0.98 (0.82-1.18)	.70	1.46 (1.10-1.92)	.01	1.39 (1.06-1.82)	.02

Abbreviations: CV, coefficient of variation; OR, odds ratio; SES, socioeconomic status.

After assessing global comparisons of readmission risk after SES adjustment, we examined specific instances where a hospital’s adjusted OR moved from significant to nonsignificant (or vice versa) after SES adjustment (data not shown). For example, when stratified by area poverty rates, the peer group classification method reduced the number of hospitals potentially eligible for penalty from 6 to 3, with 2 of these hospitals potentially becoming eligible as a result of SES adjustment. In contrast, the change in influence of female lone-parent families within the peer group–based model for quartile 1 (highest SES group) did not alter which institution became eligible for penalty. By SNH classification, the deletion of unreliable ACS estimates for poverty-adjusted AMI readmissions reduced the number of hospitals potentially eligible for penalty from 5 to 4. Similarly, the inclusion of SES adjustment for high school completion rates resulted in the deletion of 3 SNH-classified hospitals from penalty while penalizing 2 additional institutions for elevated readmission rates.

## Discussion

The use of patient SES to risk adjust hospital readmission rates remains an area of ongoing debate. However, equivalent discourse over the quality of data available for SES adjustment has been lacking. To date, there have been no fewer than 8 studies^28,29,30,31,32,33,34,35^ that have attempted to contrast hospital readmission rates against socioeconomic data derived from the ACS. However, to our knowledge, no study has attempted to disentangle its findings from the reliability of the estimates. At issue is that approximately 40% of 2015 ACS zip code poverty estimates for the entire country—the de facto scale for SES risk adjustment owing to patient privacy constraints—have CVs classified as very unreliable.^24^ Uncertainty of this magnitude jeopardizes efforts to expand risk adjustment, particularly if it results in the misclassification and mislabeling of a hospital’s performance.

Our evaluation of ACS-adjusted readmission rates reveals subtle, but significant, differences in hospital scores owing to poor precision in the data. Before the new group-based payment class, approximately 20% of SNH comparisons adjusted for high school completion rates, median household income, poverty, female lone-parent families, or area unemployment were so imprecise that they either concealed or distorted differences in readmission risk. These findings suggest a possible double jeopardy of expanding the HRRP on the basis of SNH designation: account for factors known to predispose patients to readmission in ways unrelated to the quality of care they received, but risk misclassifying hospital performance.

By comparison, there was significant improvement in estimate reliability once adjustment was derived using CMS’s new peer groups, with the percentage of variation in scores owing to estimate reliability decreasing from 20% to 3%. Although one explanation for this improvement is the shifting of hospitals into new classes based on similar proportions of dual Medicare and Medicaid payer status, these findings may also suggest that reliability improved simply because it shifted patients with more reliable data into different quartiles. Historically, the composition of lower SES neighborhoods tends to be more homogeneous, and thereby less susceptible to variation, whereas neighborhoods of greater affluence typically have more variance in income and other socioeconomic characteristics. As such, the shift of nearly 40% of SNHs into the highest SES peer groups under the new payment grouping method may be the primary reason for improved reliability in scores. Although an alternative method to increase homogeneity in the estimates is to expand the analysis to US Census Blocks or Tracts, there are 2 hurdles to this approach. First, patient zip codes, when recorded, are typically the finest geographic footprint that hospitals release. Second, and more problematic, is that the reliability of the ACS typically gets worse as the geographic area gets smaller.^36^

A twist on these findings was that, with few exceptions, the peer group–based criteria largely reduced the need for additional socioeconomic adjustment. Key exceptions were found for CHF readmissions adjusted with area poverty and high school completion rates. However, the lack of consistency in association between readmission risk and SES when these same variables were used to adjust AMI and pneumonia rates raises questions as to the sensitivity of aggregate data for representing the specific social and economic conditions widely held to be associated with health outcomes.

An additional challenge in using the ACS that was not discussed in this study is whether its reliability is suitable for annual data surveillance. It takes 5 years of surveys to build the block, tract, and zip code estimates that are released for public use. However, 80% of the data contained in the 2014 five-year estimates overlap the 2013 survey estimates.^25^ This means that annual changes in an area’s social, economic, or housing profile will often be driven by differences in data from the nonoverlapping years. Our team’s previous evaluations^37^ have shown that annual changes in ACS-reported socioeconomic conditions are more likely to arise because of sampling errors, as opposed to actual changes in living conditions. Additional tests are still needed to assess the fitness of the ACS for use on an annual basis.

In 2014, the National Quality Forum^38^ assembled a panel of 158 health care systems, consumer advocates, purchasers, and measurement developers to discuss whether to expand hospital performance and accountability measures to account for patient sociodemographic complexity. The need for additional risk adjustment was based on consistent findings that hospitals that served a disproportionately large share of Medicaid and uninsured patients (SNHs) were being penalized under CMS’s pay-for-performance programs. In some cases, they were more than twice as likely as non-SNHs to be penalized.^3^ More than 90% of the National Quality Forum’s expert panel^38^ uniformly supported recommendations to expand performance measures to include patient SES. At the time, CMS was 1 of 8 participants that opposed this point of view on the grounds that further risk adjustment would establish different standards of care for health care systems and/or mask disparities in the quality of care provided.^39^ The recent change in position by CMS speaks to the significance of accounting for the disproportionate impact of HRRP penalties on hospitals that primarily provide services to low-income populations.

Even if one agrees with expanding HRRP criteria to include other measures of patient SES, many advocates also believe that SES adjustment may have a negative effect on SNH rankings.^40^ One concern is that without upstream changes to the root cause of health inequalities, the added adjustment will force hospitals to become even more accountable to economically vulnerable patients.^40^ At issue is that SNHs already operate on a minimal or negative budget, which is often stretched because of cost imbalances resulting from the disproportionate share of reimbursement program cutbacks and revenue gained from expanded insurance coverage mandated by the Affordable Care Act.^41,42^ Another concern is that SES adjustment will “adjust away” instances when economically vulnerable patients experience treatment bias or receive treatment based on what they can afford.^43^ Until recently, this has been the point of view of CMS and one of the reasons the HRRP had yet to expand its performance criteria.^39^ Others^44,45^ contend that further adjustment is unfair because it requires holding hospitals accountable for results of care that are beyond their control. This critique becomes magnified owing to the lack of direct measurement for many of the factors deemed to be instrumental to increased risk of rehospitalization. That our findings show inconsistency in the association between SES and readmission risk across all 3 medical conditions lends support to this argument. However, it nevertheless remains difficult to substantiate many of these concerns because broader measures of social and economic conditions have yet to be included within CMS performance measures.

### Limitations

Our findings should be interpreted in light of the limitations of this study. Reliance on administrative data to conceptualize specific social processes at an individual level has well-known limitations. Nonetheless, the ACS is currently the best available resource to account for social risk factors owing to the lack of patient-level data in hospital registries. Similarly, our findings were limited to readmissions contained in a single state’s SID file. As a result, we were unable to determine whether these rates represented regional patterns that might have been further identified through additional data linkages. Although we suspect that our findings represent trends that would also appear in other states using different measures or other medical and surgical conditions, we did not determine whether our findings were an exception or rule. In addition, we did not account for whether any hospitals included in our analysis were classified as critical access hospitals. Although critical access hospitals are exempt from the HRRP, hospital readmissions are a central focus on its internal performance reports to CMS. Subsequent analyses will further address these limitations, as well as contribute new questions that can add to this discussion.

## Conclusions

Studies are now emerging about the reliability of the ACS and what is at stake for communities that are having to make planning decisions based on bad data. Issues of data uncertainty in the ACS have not yet permeated into evaluations of risk-adjusted hospital performance measurement. If the HRRP were to continue to expand its adjustment criteria to account for patient SES case mix, it is possible that some health care systems would be buried further in debt simply because data with poor estimate reliability were used to adjust their scores. Uncertainty of this magnitude jeopardizes efforts to expand risk-adjustment criteria, particularly if they incorrectly penalize or reward health care systems that may be undeserving of either. One alternative to these challenges is to improve the demographic and socioeconomic data collected from patients, but the fact remains that there is no system in the United States for such information to be systematically collected, standardized, and effectively used.
